# Inflammation, oxidative stress, glomerular filtration rate, and albuminuria in elderly men: a cross-sectional study

**DOI:** 10.1186/1756-0500-5-537

**Published:** 2012-09-27

**Authors:** Elisabet Nerpin, Johanna Helmersson-Karlqvist, Ulf Risérus, Johan Sundström, Anders Larsson, Elisabeth Jobs, Samar Basu, Erik Ingelsson, Johan Ärnlöv

**Affiliations:** 1 Department of Public Health and Caring Sciences/Geriatrics, Uppsala University, SE- 751 85, Uppsala, Sweden; 2 Department of Public Health and Caring Sciences/Clinical Nutrition and Metabolism, Uppsala University, SE-751 85, Uppsala, Sweden; 3 Department of Public Health and Caring Sciences/Oxidative stress and Inflammation, Uppsala University, SE-751 85, Uppsala, Sweden; 4 Department of Medical Sciences, Acute and Internal Medicine, Uppsala University, SE-751 85, Uppsala, Sweden; 5 Clinical chemistry, University hospital, SE-751 85, Uppsala, Sweden; 6 Uppsala Clinical Research Centre, Uppsala University, SE-752 37, Uppsala, Sweden; 7 Department of Medical Epidemiology and Biostatistics, Karolinska Institutet, SE-171 77, Stockholm, Sweden; 8 Biochimie, Biologie Moléculaire et Nutrition, Faculté de Pharmacie, Université d'Auvergne, Clermont-Ferrand, 63001, Clermont-Ferrand cedex 1, France; 9 School of Health and Social Studies, Dalarna University, SE-79188, Falun, Sweden

**Keywords:** Inflammation, Oxidative stress, Glomerular filtration rate and albuminuria

## Abstract

**Background:**

The role of inflammation and oxidative stress in mild renal impairment in the elderly is not well studied. Accordingly, we aimed at investigating the associations between estimated glomerular filtration rate (eGFR), albumin/creatinine ratio (ACR), and markers of different inflammatory pathways and oxidative stress in a community based cohort of elderly men.

**Findings:**

Cystatin C-based GFR, ACR, and biomarkers of cytokine-mediated inflammation (interleukin-6, high-sensitivity C-reactive protein[CRP], serum amyloid A[SAA]), cyclooxygenase-mediated inflammation (urinary prostaglandin F2α [PGF2α]), and oxidative stress (urinary F2 isoprostanes) were assessed in the Uppsala Longitudinal Study of Adult Men(n = 647, mean age 77 years).

**Results:**

In linear regression models adjusting for age, BMI, smoking, blood pressure, LDL-cholesterol, HDL-cholesterol, triglycerides, and treatment with statins, ACE-inhibitors, ASA, and anti-inflammatory agents, eGFR was inversely associated with CRP, interleukin-6, and SAA (β-coefficient −0.13 to −0.19, p < 0.001 for all), and positively associated with urinary F2-isoprostanes (β-coefficient 0.09, p = 0.02). In line with this, ACR was positively associated with CRP, interleukin-6, and SAA (β- coefficient 0.09-0.12, p < 0.02 for all), and negatively associated with urinary F2-isoprostanes (β-coefficient −0.12, p = 0.002). The associations were similar but with lower regression coefficients in a sub-sample with normal eGFR (>60 ml/min/1.73 m2, n = 514), with the exception that F2-isoprostane and SAA were no longer associated with eGFR.

**Conclusion:**

Our data indicate that cytokine-mediated inflammation is involved in the early stages of impaired kidney function in the elderly, but that cyclooxygenase-mediated inflammation does not play a role at this stage. The unexpected association between higher eGFR/lower albuminuria and increased F2-isoprostanes in urine merits further studies.

## Background

The increased risk of cardiovascular mortality in patients with chronic kidney disease (CKD) [1,2] has been attributed to the clustering of cardiovascular risk factors seen in these patients [1,3-5]. Untraditional cardiovascular risk factors such as oxidative stress [6,7] and inflammation [8] are more prevalent in CKD patients than in normal individuals [9], and have also been associated with adverse cardiovascular outcomes [5,10-12] and progression of renal injury in these patients [10]. These observations suggest that oxidative stress and inflammation may have an important role in the development of CKD. However, the role of inflammation and oxidative stress in mild renal impairment, particularly the role of vasoconstrctive prostaglandin F_2α_ and F_2_-isoprostanes, is not well studied. Moreover, data on the association between cytokine mediated inflammation and mild renal impairment in the elderly are scarce.

Cytokines (interleukin-6 [IL-6]), acute-phase proteins (C-reactive protein [CRP], serum amyloid A protein [SAA]), and prostaglandins (PGs) are involved in inflammatory responses and are used as indicators of systemic inflammation. Prostaglandins of the 2-series are formed from arachidonic acid by cyclooxygenases at sites of inflammation, and prostaglandin F_2α_ (PGF_2α_)—a major prostaglandin—can be reliably quantified from the stable PGF_2α_ metabolite (15-keto-dihydro-PGF_2α_) in urine [13]. F_2_-isoprostanes, which are prostaglandin derivatives, are formed by free-radical-catalysed peroxidation of arachidonic acid. 8-Iso-PGF_2α_, a major F_2_-isoprostane, is currently regarded as one of the most reliable indicators of *in vivo* lipid peroxidation and oxidative stress [14-16].

Based on previous data, we hypothesised that inflammation and oxidative stress are involved in the early stages of the development of CKD. Accordingly, we investigated cross-sectional associations between estimated glomerular filtration rate (eGFR), albuminuria (albumin/creatinine ratio [ACR]), plasma CRP, IL-6, and SAA, and urinary PGF_2α_ and F_2_-isoprostanes in a community-based sample of elderly men. Moreover, we studied two pre-specified subgroups with normal eGFR (> 60 ml/min/1.73 m^2^) and ACR (< 3 mg/mmol).

## Methods

### Study sample

The Uppsala Longitudinal Study of Adult Men (ULSAM) was started in 1970. All fifty-year-old men, born in 1920–24 and living in Uppsala, Sweden, were invited to participate in a health survey initially concentrating on identification of risk factors for cardiovascular disease (described in detail at http://www.pubcare.uu.se/ULSAM/). The present analyses are based on the fourth examination cycle of the ULSAM cohort, when subjects were approximately 77 years old (1997–2001, n = 839). Of these, 647 (77%) had valid measurements of serum cystatin C, urinary albumin/creatinine ratio, IL-6, CRP, SAA, urinary PGF_2α_, F_2_-isoprostanes, and covariates. All participants gave written informed consent and the Ethics Committee of Uppsala University approved the study protocol.

### Clinical and biochemical evaluation

Serum cystatin C, high-sensitivity CRP, SAA, and urine albumin were measured using a BN ProSpec nephelometer (Siemens, Deerfield, IL, USA). The total analytical imprecision of the cystatin C assay was 4.8% at 0.56 mg/L and 3.7% at 2.85 mg/L High-sensitivity IL-6 was analysed with an ELISA kit (IL-6 HS; R&D Systems, Minneapolis, MN, USA). eGFR was calculated from serum cystatin C results in mL/min/1.73 m^2^ by the formula y = 77.24x^-1.2623^, which have been shown to be closely correlated with iohexol clearance [17].

Urine creatinine was analysed by a modified kinetic Jaffe reaction on an Architect Ci8200® analyser (Abbott, Abbot Park, IL, USA) and reported in mmol/L; creatinine-related urine albumin was calculated from the Prospec® results. Urinary samples were analysed for 15-keto-dihydro-PGF_2α_ , a stable metabolite of PGF_2α_, with a radioimmunoassay that has been described previously in detail [18]. Urinary 15-keto-dihydro-PGF_2α_ concentrations were divided by urinary creatinine levels. Urinary F_2_-isoprostanes (free 8-iso-PGF_2α_ without any prior extraction or purification) were analysed with a radioimmunoassay that has been described previously [19]. Urinary 8-iso-PGF_2α_ concentrations were divided by urinary creatinine levels.

Plasma glucose, serum lipids, blood pressure, and body mass index (BMI) were assessed as previously described [20]. Diabetes mellitus was diagnosed as a fasting plasma glucose level of ≥ 7.0 mmol/l (≥ 126 mg/dl), or by the use of oral hypoglycaemic agents or insulin. Smoking status (current smoker or non-smoker) and information concerning pharmacological treatment was recorded using a questionnaire. Information about hospitalisation because of myocardial infarction, angina pectoris, ischaemic stroke, and heart failure was obtained from the Swedish hospital discharge register.

### Statistical analysis

Logarithmic transformation was performed to obtain a normal distribution of urine albumin/creatinine ratio, CRP, PGF_2α_, IL-6, SAA, F_2_-isoprostanes, glucose, and triglycerides. All other variables were normally distributed. Linear regression analyses were used to assess the cross-sectional associations between CRP, PGF_2α_, IL-6, SAA, and F_2_-isoprostanes (independent variables) and eGFR and albumin/creatinine ratio (dependent variables in separate models). We used the directed acyclic graphs (DAG) approach to establish a parsimonious model with minimised confounding of the effect estimates in the statistical model B.

The following models were performed:

Model A: age-adjusted (continuous);

Model B: age (continuous), BMI (continuous), smoking (binary), systolic and diastolic blood pressure (continuous), LDL-cholesterol, HDL-cholesterol, and triglycerides (continuous), statin treatment (binary), ACE inhibitors, ASA, and anti-inflammatory agents including cortisone (binary).

We also performed the above analyses in a subgroup analysis between CRP, PGF_2α_, IL-6, SAA, and F_2_-isoprostanes in the following pre-specified subgroups: (1) participants with eGFR > 60 ml/min/1.73 m^2^ (n = 514), and (2) participants with ACR < 3 mg/mmol (n = 522).

Moreover, we performed analyses in participants without anti-inflammatory agents (n = 612). We also performed a secondary multivariable model where diabetes and cardiovascular disease where added to multivariable model B (model C). The reason that diabetes and cardiovascular disease was not included in our primary models was that these factors may be considered to be in the causal pathway between inflammation and renal damage. Moreover, we investigated the association between eGFR and F_2_-isoprostanes after excluding participants with diabetes (n = 83) and individuals with the highest eGFR (upper decile, > 95 ml/min/1.73 m^2^, n = 63) to rule out the risk of glomerular hyperfiltration as an explanation of our findings.

In our primary analysis, we modelled CRP, PGF_2α_, IL-6, SAA, F_2_-isoprostanes, eGFR, and albumin/creatinine ratio as continuous variables (expressed as a 1-standard deviation increase).

A two-sided p-value of < 0.05 was regarded as significant in all analyses. The statistical software package STATA 11.0 was used (Stata Corp., College Station, TX, USA).

## Findings

Table 1 shows the characteristics of the study population.

**Table 1:** Characteristics of the whole study population

**Variable**	**Whole sample (n = 647)**
Age (years)	77.5 ± 0.8
Urine albumin creatinine ratio (mg/mmol)	4.6 ± 19.4
Serum cystatin C (mg/L)	1.09 ± 0.28
eGFR* (ml/min/1.73 m2)	73.6 ± 17.4
Fasting plasma glucose (mmol/L)	5.9 ± 1.3
Systolic blood pressure (mmHg)	150.9 ± 20.7
Diastolic blood pressure (mmHg)	81.3 ± 9.7
Body mass index (kg/m2)	26.3 ± 3.5
Serum triglyceride (mmol/L)	1.4 ± 0.6
HDL cholesterol (mmol/L)	1.3 ± 0.3
LDL cholesterol (mmol/L)	3.5 ± 0.9
Urine 15-keto-dihydro-PGF2α (nmol/mmol)	0.32 ± 0.18
Serum interleukin-6 (ng/L)	3.9 ± 2.7
Serum amyloid A protein (mg/L)	11.3 ± 43.6
high sensitivity C-reactive protein (mg/L)	3.8 ± 6.8
Urine F2-isoprostane (nmol/mmol)	0.20 ± 0.10
Diabetes mellitus – n (%)	91 (14.1)
Smoking – n (%)	45 (7.0)
Dyslipidemia – n (%)	226 (34.9)
Lipid lowering treatment – n (%)	119 (18.4)
Cardiovascular disease – n (%)	175 (27.1)
Hypertension – n (%)	292 (45.1)
Antihypertensive treatment – n (%)	272 (42.0)
ACE-inhibitor – n (%)	109 (16.9)
ASA medicine – n (%)	193 (29.8)
Corticosteroid treatment – n (%)	26 (4.0)
Non-steroidal anti-inflammatory drugs – n (%)	35 (5.4)

Data are mean ± SD for continuous variables and n. (%) for dichotomous variables. *eGFR was estimated from cystatin C.

### Associations between inflammatory biomarkers, cystatin C-based glomerular filtration rate (eGFR), and albumin/creatinine ratio levels (ACR)

In the whole cohort, eGFR was inversely associated and ACR was positively associated with CRP, IL-6, SAA, when adjusting for age, BMI, smoking, systolic and diastolic blood pressure, hypertension treatment, LDL-cholesterol, HDL-cholesterol, triglycerides, treatment with statin, ACE inhibitors, ASA, anti-inflammatory agents, diabetes, and previous cardiovascular disease (models A–C, Table 2).

**Table 2:** **Cross-sectional associations between high sensitive C-Reactive Protein (CRP), Interleukin 6 (IL- 6), Prostaglandin F**_**2 **_**alpha (PGF**_**2**_**alpha), serum amyloid protein (SAA) and eGFR**_**cyst**_**and albumin creatinine ratio (ACR) at age 77: Multivariable regression**

	**Cystatin C estimated glomerular filtration rate**		**lnAlbumin creatinine ratio (ACR)**	
	**total cohort (n = 647)**	**eGFR > 60 ml/min/1.73 m**^**2**^**(n = 514)**	**total cohort (n = 647)**	**ACR < 3 mg/mmol (n = 522)**
	**β-coefficient (95% CI)**	**β-coefficient (95% CI)**	**β-coefficient (95% CI)**	**β-coefficient (95% CI)**
**Model A**				
*ln* CRP (mg/L)	−0.23 (−0.30 to −0.15)***	−0.10 (−0.17 to −0.34)**	0.11 (0.03 to 0.19) **	0.07 (−0.04 to 0.06)
*ln* PGF_2α_ (nmol/mmol)	−0.01 (−0.07 to 0.08)	−0.01(−0.08 to −0.05)	0.01 (−0.07 to 0.09)	0.05 (0.002 to 0.10) *
*ln* IL-6 (ng/L)	−0.28 (−0.35 to −0.20)***	−0.10 (−0.17 to −0.04) **	0.13 (0.06 to 0.21) **	0.01 (−0.04 to 0.05)
*ln* SAA (mg/L)	−0.15 (−0.22 to-0.07)***	−0.05 (−0.12 to 0.02)	0.14 (0.06 to 0.21) **	0.05 (0.002 to 0.10) *
**Model B** (DAG adjusted)				
*ln* CRP (mg/L)	−0.19 (−0.26 to −0.11)***	−0.09 (−0.16 to −0.02) **	0.09 (0.01 to 0.16) *	−0.01 (−0.05 to 0.04)
*ln* PGF_2α_ (nmol/mmol)	−0.01 (−0.07 to 0.08)	−0.02 (−0.09 to 0.04)	0.01 (−0.06 to 0.09)	0.03 (−0.01 to 0.08)
*ln* IL-6 (ng/L)	−0.23 (−0.30 to −0.15) ***	−0.09 (−0.16 to −0.02)*	0.11 (0.03 to 0.19) **	−0.01 (−0.05 to 0.04)
*ln* SAA (mg/L)	−0.13 (−0.21 to −0.06)**	−0.05 (−0.12 to 0.02)	0.12 (0.05 to 0.20) **	0.04 (−0.008 to 0.09)
**Model C**				
*ln* CRP (mg/L)	−0.19 (−0.26 to −0.11)***	−0.09 (−0.16 to −0.02)**	0.08 (0.01 to 0.16) *	−0.01 (−0.06 to 0.04)
*ln* PGF_2α_ (nmol/mmol)	−0.01 (−0.07 to 0.08)	−0.02 (−0.09 to 0.04)	−0.04 (−0.08 to 0.07)	0.03 (−0.02 to 0.08)
*ln* IL-6 (ng/L)	−0.23 (−0.30 to −0.15)***	−0.09 (−0.16 to −0.02)**	0.09 (0.01 to 0.16) *	−0.01 (−0.06 to 0.04)
*ln* SAA (mg/L)	−0.13 (−0.21 to −0.06)***	−0.05 (−0.12 to 0.02)	0.11 (0.04 to 0.19) **	0.04 (−0.01 to 0.09)

Data are regression coefficients for a 1-SD higher ln C-reactive protein (CRP), ln interleukin 6 (IL- 6), ln prostaglandin F_2_ ln α (PGF_2α_),ln serum amyloid protein A (SAA). Model A was adjusted for age; model B was adjusted according to directed acyclic graphs (DAG): age, BMI, smoking, systolic and diastolic blood pressure, LDL, HDL, and triglyceride, statin treatment, ACE inhibitor-, ASA-, anti-inflammation-, and cortisone medication. Model C was adjusted for: age, BMI, smoking, systolic and diastolic blood pressure, hypertension treatment, LDL, HDL, and triglyceride, statin treatment, diabetes, ACE inhibitor-, ASA-, anti-inflammation-, and corticosteroid treatment, and CVD. * p < 0.05, **p < 0.01. *** p < 0.001.

After further exclusion of participants with impaired eGFR (< 60 ml/min/1.73 m^2^) the association between eGFR, CRP, and IL-6, remained statistically significant in all models but with lower regression coefficients. No significant association was seen between eGFR and urinary PGF_2α_ in the whole cohort or in participants with eGFR > 60 ml/min/1.73 m^2^. After exclusion of participants with ACR > 3 mg/mmol, ACR was found to be positively associated with PGF_2α_ metabolite and SAA adjusted for age (Table 2), while no significant association was seen in models B and C between ACR and the inflammatory markers. The results were unaltered in a sub-sample of participants without anti-inflammatory agents (data not shown).

### Association between oxidative stress, glomerular filtration rate, and albumin/creatinine ratio levels

In the whole cohort, eGFR was positively associated and ACR was inversely associated with urinary F_2_-isoprostanes in all multivariable models (models A–C, Table 3). After further exclusion of participants with impaired eGFR (< 60 ml/min/1.73 m^2^) and ACR > 3 mg/mmol separately, no associations were found between levels of the two kidney markers and urinary F_2_-isoprostanes.

**Table 3:** **Cross-sectional associations between oxidative stress (urinary F**_**2**_**-Isoprostanes) and eGFR**_**cyst **_**and albumin creatinine ratio (ACR) at age 77: Multivariable regression**

	**Cystatin c estimated glomerular filtration rate**		**lnAlbumin creatinine ratio (ACR)**	
	**total cohort (n = 647)**	**eGFR > 60 ml/min/1.73 m**^**2**^**(n = 514)**	**total cohort (n = 647)**	**ACR < 3 mg/mmol (n = 522)**
	**β-coefficient (95% CI)**	**β-coefficient (95% CI)**	**β-coefficient (95% CI)**	**β-coefficient (95% CI)**
**Model A**				
*ln* F2-isoprostane (nmol/mmol)	0.08 (0.006 to 0.16)*	0.04 (−0.03 to 0.11)	−0.13 (−0.20 to −0.05) ***	0.004 (−0.04 to 0.05)
**Model B** (DAG adjusted)				
*ln* F2-isoprostane (nmol/mmol)	0.09 (0.02 to 0.17)*	0.03 (−0.03 to 0.10)	−0.12 (−0.19 to −0.04) **	0.0008 (−0.05 to 0.05)
**Model C**				
*ln* F2-isoprostane (nmol/mmol)	0.09 (0.01 to 0.16)*	0.03 (−0.04 to 0.10)	−0.12 (−0.20 to −0.05) ***	−0.001 (−0.05 to 0.05)

Data are regression coefficients for a 1-SD urinary ln F2-isoprostanes. Model A was adjusted for age; model B was adjusted according to directed acyclic graphs (DAG): age, BMI, smoking, systolic and diastolic blood pressure, LDL, HDL, and triglyceride, statin treatment, ACE inhibitor-, ASA-, anti-inflammation-, and cortisone medication. Model C was adjusted for: age, BMI, smoking, systolic and diastolic blood pressure, hypertension treatment, LDL, HDL, and triglyceride, statin treatment, diabetes, ACE inhibitor-, ASA-, anti-inflammation-, and corticosteroid treatment, and CVD. * p < 0.05, **p < 0.01. *** p < 0.001.

The association between eGFR and F_2_-isoprostanes was essentially similar after excluding participants with diabetes and eGFR > 95 ml/min/1.73 m^2^ (model B, β-coefficient 0.08 [95% CI 0.005–0.15]; p = 0.04).

## Discussion

### Principal findings

In this community-based sample of elderly men, reduced eGFR and increased ACR were associated with higher systemic CRP, IL-6, and SAA concentrations. The association between eGFR, CRP, and IL-6 remained significant in participants without any apparent signs of kidney dysfunction (eGFR > 60 ml/min/1.73 m^2^).

Our findings are in accordance with most, but not all [21], previous community-based studies that found independent associations between biomarkers of cytokine-mediated inflammation (C-reactive protein, tumor necrosis factor alpha, interleukin-6, and fibrinogen) and eGFR, measured with serum creatinine [22-25], cystatin C [8,26], and albuminuria [25,27,28]. We are aware of one previous study that have reported these associations in elderly individuals without any apparent signs of kidney damage or dysfunction [8]. Systemic inflammation has been considered to be a risk factor for CKD, but may also represent a common pathway by which cardiovascular risk factors interact to amplify renal injury [9,29].

To our knowledge, this is the first study to have investigated the association between markers of kidney damage and dysfunction and *in vivo* PGF_2α_ concentrations. However, no independent associations were seen between these markers of kidney pathology and urinary PGF_2α_ metabolite in this study, indicating that cyclooxygenase-mediated inflammation is not involved in the early stages of chronic kidney disease.

Surprisingly, increased eGFR and reduced ACR were associated with higher levels of urinary F_2_-isoprostanes in the whole cohort. Since oxidative stress is suggested to play an important role in the development of kidney disease, based on experimental studies [30,31], this finding was contradictory to what we originally hypothesised. Yet, a similar finding was seen in a recent study from the Framingham Offspring Study, where individuals with CKD had lower urinary isoprostanes than individuals without CKD [25]. In contrast, a study on obese children [32] failed to show any linear correlation between plasma cystatin C, albuminuria, and urinary F_2_-isoprostanes.

The unexpected associations found between kidney biomarkers and intact urinary F_2_-isoprostanes in the present study may possibly be related to the fact that F_2_-isoprostanes were quantified in urine but not in plasma [33]. Studies have shown that patients with manifested moderate to severe chronic kidney disease [9] and dialysis patients [6,7,29,34] have higher plasma concentrations of F_2_-isoprostane than healthy subjects. Furthermore, an inverse correlation was shown between eGFR and plasma F_2_-isoprostanes in hypertensive patients [29]. The kidney is one of the major sites of both 15-prostaglandin dehydrogenase (15-PGDH) and Δ^13^-reductase, the two major enzymes that metabolise prostaglandins and F_2_-isoprostanes to 15-keto-dihydro-metabolites for further degradation to more polar β- and ω-oxidised products in the liver before excretion together with unmetabolised primary PGF_2α_ and F_2_-isoprostanes [35,36]. Perhaps the discrepancy in plasma and urine may be explained by the possibility that impairment of these enzymes in the kidney affects both the metabolism and further excretion of intact F_2_-isoprostanes into the urine, although to our knowledge this has not been reported. The fact that the positive association between eGFR and urinary F_2_-isoprostanes was essentially similar after excluding participants with diabetes and individuals with the highest eGFR indicates that glomerular hyperfiltration does not explain the unexpected findings.

The strengths of our investigation include the homogenous, community-based study sample with detailed characterisation of glucometabolic variables, cardiovascular risk factors, and lifestyle factors. Some limitations of our study must also be discussed. As we only examined men of the same age and of similar ethnic background, the degree of generalizability to women or to other ages and ethnic groups is unknown. Furthermore, we did not use the gold standard method to measure GFR (clearance measurements with exogenous substances), as this method is generally not feasible in large study samples. Also, our cystatin C assay was not calibrated to the new international reference standard [37,38]. Moreover, as no data on serum creatinine was available, we were unable to use the recently proposed GFR equation that incorporates both calibrated cystatin C and creatinine [39]. However, the cystatin C-based GFR equation used in the present study [17] has been shown to be closely associated with GFR measured with iohexol clearance also in individuals with GFR in the normal range. This study was also limited by the use of a single urine collection for assessment of ACR. Yet, any potential bias from variations in ACR and GFR levels would most likely conservatively bias our regression estimates. Moreover, no conclusions regarding causality should be drawn from our cross-sectional observational data. Finally, we cannot rule out that participants with unidentified inflammatory disease may have influenced the associations, but as this is a healthy community based-sample, this potential bias is not likely to be major.

## Conclusions

Our data indicate that cytokine-mediated inflammation is involved already in the early stages of impaired kidney function in the elderly, but that cyclooxygenase-mediated inflammation does not appear to play a role at this stage. Whether anti-inflammatory therapies are effective in slowing down the deterioration of kidney function in the elderly remain to be established. In order to clarify the relevance and the underlying pathophysiology of the unexpected association between higher urinary F_2_-isoprostane concentrations and higher eGFR/lower albuminuria, further experimental studies are needed.

## Competing interests

The authors declare that they have no competing interests.

## Authors' contributions

Conception or design, or analysis and interpretation of data. Drafting of the article or revising it. Providing intellectual content of critical importance to the work described. Final approval of the version to be published. All authors read and approved the final manuscript.
